# Sex-Specific Risk Factors for Short- and Long-Term Outcomes after Surgery in Patients with Infective Endocarditis

**DOI:** 10.3390/jcm11071875

**Published:** 2022-03-28

**Authors:** Christine Friedrich, Mohamed Salem, Thomas Puehler, Bernd Panholzer, Lea Herbers, Julia Reimers, Lars Hummitzsch, Jochen Cremer, Assad Haneya

**Affiliations:** 1 Department of Cardiovascular Surgery, University Hospital Schleswig-Holstein, Campus Kiel, 24105 Kiel, Germany; mohamed.salem@uksh.de (M.S.); thomas.puehler@uksh.de (T.P.); bernd.panholzer@uksh.de (B.P.); lea.herbers@uksh.de (L.H.); julia.reimers@uksh.de (J.R.); jochen.cremer@uksh.de (J.C.); assad.haneya@uksh.de (A.H.); 2 Department of Anaesthesiology and Intensive Care Medicine, University Medical Center Schleswig-Holstein, Campus Kiel, 24105 Kiel, Germany; lars.hummitzsch@uksh.de

**Keywords:** infective endocarditis, sex-specific, gender, risk factors, survival

## Abstract

(1) Background: Surgery for infective endocarditis (IE) is associated with considerable mortality and it is controversial whether the female gender is predictive for a worse outcome. This large single-center study investigated the impact of sex on outcomes after surgery for IE. (2) Methods: 413 patients (25.4% female) were included into this retrospective observational study. Univariate and multivariable analyses identified sex-specific risk factors for 30 day and late mortality. Survival was estimated by the Kaplan-Meier-method. (3) Results: Women presented more often with mitral valve infection (*p* = 0.039). Men presented more frequently with previous endocarditis (*p* = 0.045), coronary heart disease (*p* = 0.033), and aortic valve infection (*p* = 0.005). Blood transfusion occurred more frequently intraoperatively in women (*p* < 0.001), but postoperatively in men (*p* = 0.015) and men had a longer postoperative stay (*p* = 0.046). Women showed a higher 30 day mortality than men (*p* = 0.007) and female gender was predictive for 30 day mortality (OR 2.090). Late survival showed no sex-specific difference (*p* = 0.853), and the female gender was not an independent predictor for late mortality (*p* = 0.718). Risk factors for early and late mortality showed distinct sex-specific differences such as increased preoperative CRP level in women and culture-negative IE in men.

## 1. Introduction

Infective endocarditis (IE) is a rare but severe disease with a higher incidence in men and a male/female ratio ranging mostly from 1.3:1 to 3:1 in hospital-based studies [1,2,3,4,5]. Though the causes for this sex-specific difference are not fully understood, a higher rate of pre-disposing heart conditions in men [5,6] may contribute to a lower incidence of IE in women.

Early diagnosis and therapy are essential to reduce morbidity and mortality in patients suffering from IE [7]. Surgical treatment was carried out in about 50% of cases in the European infective endocarditis registry [3], but women underwent cardiac surgery for IE less frequently than men in several studies [4,8,9].

Despite major medical advances in diagnostics and therapy, IE is still associated with severe morbidity and a high early mortality of around 20% [3,10,11]. In cases of IE, female gender shows no protective effect, since several studies demonstrate a similar or higher early mortality when compared to men [8,12,13,14,15]. The persistently poor overall prognosis regarding hospital mortality is, besides the female gender, attributed to several possible causative factors such as the increase of elderly and more severely ill patients, previous cardiac surgery, an increasing rate of IE in prosthetic heart valves and devices, cerebral complications, renal failure, preoperative ventilation, New York Heart Association heart failure (NYHA) stage, paravalvular abscess, *S. aureus* infection, and withholding of indicated surgery [3,10,12,16]. However, there are controversial implications if the female gender is an independent predictor for mortality after surgery for IE [4,13,16,17]. Older age, a different spectrum of comorbidities, and a lower rate of surgical treatment are discussed as risk factors for a poorer outcome in women [8,9,12]. Sex-specific analysis revealed older age, preoperative dialysis, identification of the endocarditis focus [12], and a poor response to antibiotics [2] as independent risk factors in female patients. Likewise, studies on mid- or long-term outcome provide conflicting results. While some studies revealed a lower survival rate in women [9,12], others found a similar survival rate [18], and in several studies the female gender was no independent predictor for late mortality [8,9,17,19].

Sex-specific subgroup analyses on adjusted risk factors for early and late mortality after surgery for IE are scarce, therefore we analyzed predictors for early and late mortality for women and men separately to detect underlying causes for their different outcomes.

## 2. Materials and Methods

### 2.1. Patients and Study Design

This retrospective observational single-center study included 413 consecutive patients ≥ 18 years, 105 (25.4%) women and 308 (74.6%) men, who were operated on for IE between January 2002 and February 2020 in our department.

Pre-, intra-, and post-operative findings of women and men and their risk factors for mortality were analyzed and compared. Data were collected from the institution’s database and patient records. The primary endpoints were 30-day mortality and survival during follow-up; secondary endpoints were pre- and peri-operative details, post-operative outcome, and sex-specific risk factors for early and late mortality. All-cause survival during follow-up was obtained by inquiries at the registry offices. The number of patients in the intraoperative course and during the follow-up is shown in Supplementary Figure S1. Active IE was defined as patients receiving antibiotic therapy at the time of admission. The study protocol was approved by the local Ethics Committee and informed patient consent was obtained at primary hospital stay.

Preliminary sub-results of this study without sex-specific risk factor analysis were published in German [20].

### 2.2. Patient Management

A transthoracic echocardiogram (Vivid E9, General Electric Company, Boston, MA, USA) was performed on every patient, recording the location and size of vegetations, the presence of valve destruction or abscess, as well as left ventricular ejection fraction (LVEF). The antibiotic treatment started immediately after diagnosis of IE and an intravenous treatment regimen was initiated for at least 4–6 weeks independently of the time of surgery. Blood cultures were obtained from all patients to identify the pathogenic organisms and their sensitivities to medical treatment. All patients admitted with stroke underwent brain scan computer tomography to exclude any risk of bleeding prior to surgery and also to get a prognosis for intubated patients and coma patients. A neurologist was consulted to evaluate the neurological findings. The indication for the operation was made after interdisciplinary discussion between cardiologists and cardiac surgeons on the basis of the currently valid guidelines.

### 2.3. Surgical Procedure

Women and men with IE underwent curative surgery performed exclusively by senior surgeons. Cardiopulmonary bypass was performed by direct cannulation of the ascending aorta. In cases of aortic valve endocarditis, venous drainage was carried out through direct cannulation of the right atrium, while double cannulation of the superior and inferior vena cava was performed in cases of mitral or tricuspid valve endocarditis, with subsequent cross-clamping of ascending aorta. The decision for biological or mechanical prosthesis or valve repair was made depending on the age of the patients, the intraoperative findings, and the extent of valve destruction, as well as the patient’s preference and their compliance with long-term anticoagulation. Transesophageal echocardiography (GE Vivid E95, General Electric Company, Boston, MA, USA) was performed for assessment after surgical repair and to control the presence of residual air in the left side of the heart during rewarming. The operative technique has been described in more detail in previous papers [21].

### 2.4. Statistical Analysis

Pre-, intra-, and post-operative findings of women and men and of 30 day survivors and non-survivors were compared by univariate statistics. Continuous variables were assessed for normal distribution by the Kolmogorov-Smirnov-Test and are presented as median with range or interquartile range as appropriate, and compared by the Mann-Whitney-U-Test. Categorical variables were shown as absolute frequencies (*n*) and simple percentages and were compared by the Chi-squared or Fisher’s exact test as appropriate. Missing data were excluded pairwise and variables with missing data > 5% are indicated in the tables.

Preoperative variables with univariate association to 30 day mortality (*p* ≤ 0.1) were assessed for their adjusted impact on early mortality by multivariable logistic regression for the overall group, as well as for women and men separately, with a goodness of fit, described by Cox and Snell R-Squared, of 0.214, 0.377, and 0.231, respectively. Age was categorized as sex-specific according to the highest impact on 30 day mortality. Although EuroSCORE I and II (The European System for Cardiac Operative Risk Evaluation) [22,23] showed a significant association with mortality, we excluded it from the multivariable analyses since it complicated the detection of single risk factors due to multicollinearity. Sex-specific interaction was assessed by logistic regression analysis.

Follow-up outcome was defined as all-cause mortality of patients who survived 30 days postoperatively. Survival was estimated by the Kaplan-Meier method for right censored data and analyzed for sex-specific differences by the log rank-test. Risk factors for mortality during follow-up were assessed by Cox proportional hazards regression with forward selection for the groups separately and then included into the final models. Age of the overall group was categorized to ≥65 years for multivariable analysis based on the median value of 64 years.

All tests were conducted as two-sided and a *p*-value of ≤0.05 was assumed to be statistically significant. Statistical analysis was performed using the IBM SPSS Statistics software (version 26.0 and 28.0).

## 3. Results

**Figure 1:**
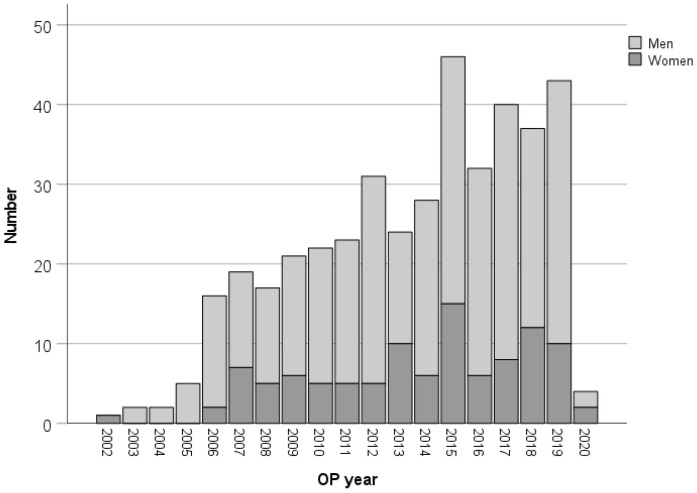
Number of patients during the study period.

The number of patients per year with surgical treatment of infective endocarditis increased over the study period (Figure 1).

### 3.1. Demographics and Clinical Details of the Study Population

Men were affected almost three times as often as women (74.6 vs. 25.4%, Table 1) and female patients with IE were only slightly older than male patients (65 vs. 64 years, *p* = 0.082). Men presented more often with coronary heart disease (46.3 vs. 34.3%, *p* = 0.033), with previous surgically treated IE (16.6 vs. 8.6%, *p* = 0.045) and with isolated infection of the aortic valve (34.7 vs. 20.0%, *p* = 0.005).

Women presented more often with previous aortic valve replacement (22.9 vs. 14.6%, *p* = 0.050), isolated infection of the mitral valve (29.5 vs. 19.8%, *p* = 0.039), and of the tricuspid valve combined with infection of other valves (7.6 vs. 2.6%, *p* = 0.036). A significant association was shown between preoperative embolization and neurological complications preoperative for both women and men (Chi^2^ *p* < 0.001).

Intraoperatively, no significant sex-specific differences regarding procedural times were observed, but women received more blood transfusions (red blood cell concentrates, 4 (0–14) vs. 2 (0–27), *p* < 0.001). Men more often underwent aortic valve surgery (78.1 vs. 62.9%, *p* = 0.002), had a higher need for blood transfusion postoperatively (2 (0–27) vs. 2 (0–17), *p* = 0.015), and a longer postoperative stay (10 vs. 9 days, *p* = 0.046; Table 2 and Table 3). In-hospital mortality tended to be higher in women (22.3 vs. 14.7%, *p* = 0.070), while 30 day mortality was significantly higher when compared to mortality in men (26.7 vs. 14.9%, *p* = 0.007).

### 3.2. Univariate Association to 30-Day Mortality

Though being without significance in the total group, sex-specific analyses revealed a significant association for pulmonary hypertension, diabetes mellitus Type 1 and 2, embolization of several organs, time from antibiotic start to surgery < 7 days, culture negative IE, and combined surgery in the male group. Only in the female group did peripheral arterial disease (PAD) show a significant association to 30 day mortality, which was confirmed by interaction analysis (Supplementary Table S1). A univariate subgroup analysis based on the overall cohort revealed that culture-negative cases had an antibiotic treatment before surgery > 7 days (62 (55.9%) less frequently when compared to culture-positive cases (202 (68.2%), *p* = 0.020). Additional sex-specific association is shown in Table S1.

### 3.3. Independent Predictors for 30 Day Mortality

In the overall group, female gender was an independent predictor for 30 day mortality (Table 4). Acute or chronic dialysis was revealed as a risk factor for early mortality in women and men. In women only, age ≥ 65 years, preoperative transfer from the intensive care unit (ICU), and increased C-reactive protein (CRP) level were risk factors. In men, age ≥ 70 years, body mass index (BMI), pulmonary hypertension, NYHA IV, cardiogenic shock, fever until surgery, culture negative IE, abscess, and embolization of several organs were predictors for early mortality.

### 3.4. Survival Analysis

**Figure 2:**
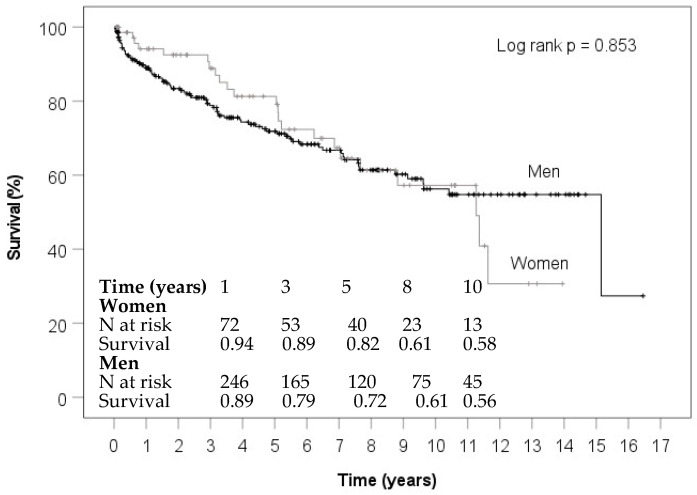
Survival of women and men during follow-up.

Median follow-up time was 3.9 (1.2; 7.7) years. Crude survival showed no sex-specific difference (*p* = 0.853, Figure 2) and women and men showed a cumulative survival of 82% vs. 72%. after 5 years and 61% in both groups after 8 years.

### 3.5. Risk Factors for Long-Term Survival

Female gender did not prove to be a risk factor for mortality during follow-up (*p* = 0.718, Table 5).

The Cox regression revealed sex-specific risk factors for late mortality. Age ≥ 70 years, arterial hypertension, LVEF < 30%, PAD, combined valve surgery, cardiogenic shock, preoperative stroke, tumor, liver disease, and abscess were identified as risk factors in the male group. Risk factors in the female group were age ≥ 65 years, NYHA IV, coronary three-vessel disease, emergency admission, diagnosis until surgery > 7 days, Staphylococcus epidermidis as a pathogenic microorganism, and preoperatively increased CRP (mg/L) level, while preoperative acute or chronic dialysis was evident in both groups.

## 4. Discussion

In this study, women showed a considerably higher early mortality when compared to men and the female gender was an independent predictor for 30 day mortality, while no significant difference was observed regarding mortality during follow-up. Independent risk factors for early and late mortality showed substantial sex-specific differences and differed strikingly from the results of the overall group.

In contrast to the review of Slipczuk et al. 2013 [10], who found a proportional increase in male IE patients from the 1970s to the 2000s, we could not show a further temporal trend in the male/female ratio during our study period from 2002 to 2020. The proportion of our surgically treated female patients was 25.4%, which is lower than 31.1% in the European infective endocarditis registry, which also included non-surgically treated patients [3]. This points to an underlying lower rate of referral to cardiac surgery in our female patients, a finding already described in several recent studies [3,9,13,15].

In contrast to previous findings [8,9,12,14], female patients in our study were not distinctly older and did not present with more comorbid conditions when compared to men. As shown in previous studies [9,13,17], IE affected the aortic valve in men most frequently, while in female patients, a predominant affection of the mitral valve was found.

Risk stratification by logistic EuroSCORE and EuroSCORE II showed a tendency towards a higher postoperative risk in female patients. However, particularly EuroSCORE II did not adequately predict the considerably higher mortality in women when compared to men. The EuroSCORE includes female gender as a risk factor, but it is based on a mixed female and male population and therefore does not take into account the different weighting of risk factors in men and women identified in previous studies [2,12] and confirmed by our results.

Despite comparable intraoperative procedural times, women received more blood transfusions than men. Higher transfusion rates and lower preoperative hemoglobin levels in women were already described for cardiac surgery [24,25], and may explain the observed intraoperative discrepancy in our study. Men tended to develop a delirium more often than women, as we already observed at aortic surgery [26]. This is in line with the findings of Wang et al. that male gender is an important predictor for postoperative delirium following cardiac surgery [27]. Moreover, red blood cell transfusion, which was more frequent in our male patients postoperatively, was shown to be an important initiating risk factor for delirium after cardiac surgery in a review by Koster et al. [28], as well as the association with prolonged postoperative stay in our male patients [29].

The overall early mortality of 17.9% in our study was comparable to the 17% mortality reported in the European endocarditis registry [3] and 17.2% mortality after surgery for IE of the aortic valve [12]. Women in our study experienced a significantly higher early mortality when compared to men as demonstrated previously [12,13,17]. However, in contrast to these studies, we found no major sex-specific differences regarding baseline clinical presentation, comorbidities, or pathogenic organisms that could explain the poorer outcome in women, but rather a gender-specific impact of risk factors, as the univariate and multivariable analysis indicated.

Weber et al. [17] stated that the severity of presentation, but not female gender, accounts for a worse outcome after IE. Contrary to their results, which included also postoperative factors and EuroSCORE II, female sex proved to be an independent predictor for early mortality in our study on preoperative predictors, confirming previous results [4,12,16]. The multivariable analysis, moreover, revealed distinct sex-specific predictors for early mortality. In female patients, an age ≥ 65 years, dialysis, preoperative transfer from the ICU, and increased CRP level were predictive. Elevated baseline CRP levels were predictive for early mortality after IE in a prospective hospital-based analysis [30], however, it was a risk factor only in our female surgical patients. Dialysis was shown to be a risk factor in women [12], but was confirmed by our study as being a risk for both genders as demonstrated recently [2]. A predominant affection of the mitral valve as identified in our female patients may be associated with a lower referral to early valve surgery in women [19], whereas affection of the aortic valve was identified as predictor for early valve surgery [19]. The protective effect of aortic valve IE identified in our female patients may support this theory of a possible earlier referral of women with aortic valve IE in a less severe preoperative condition. However, this observation should be further clarified. Only in the male group, age ≥ 70 years, BMI, pulmonary hypertension, and factors related to severity of IE, namely, fever until surgery, embolization of several organs, and culture-negative IE, were risk factors for 30 day mortality. The frequency of culture-negative IE was similar in women and men in our study but was overall higher (27.5%) when compared to the findings of the European endocarditis registry, which reports a frequency of 21% [3]. A recent study by Salsano et al. [31] showed that culture-negative IE is associated with a significantly higher adjusted postoperative risk for early mortality. Culture-negative IE may complicate treatment and result in a worse outcome, as observed in our male patients.

Unadjusted all-cause survival was similar between genders. Once the critical early period is overcome, women and men therefore seemed to have a comparable prognosis, as also shown in a previous hospital-based study [18]. In contrast, Dohmen et al. [12] and Weber et al. [17] found a significantly worse survival after surgery for IE in women when compared to men during a 12 year and a 1.76 year follow-up, respectively. A higher age and more severe degree of IE may explain the worse outcome in in these studies.

Previous studies demonstrated that female gender was not an independent predictor for mid-term and long-term mortality [8,9,17,19], which was confirmed by our analysis. Our multivariable analysis revealed distinct gender-specific risk factors for mortality during follow-up, except for age and preoperative dialysis, which were identified as risk factors in both genders, confirming the results of Dohmen et al. [12]. The meta-analysis of Liang et al. [32] indicated that a surgical treatment within one week after diagnosis was associated with lower in-hospital mortality; however, a time interval > 7 days was an independent predictor for follow-up mortality in female patients in our study, but not for early mortality, and, moreover, it did not prove as a risk factor for male patients.

This study has several limitations. The retrospective single-center design carries a possible risk for unknown confounders due to unregistered variables. The limited sample size of the female cohort decreases the statistical power and may have impaired the detection of risk factors in the female group.

## 5. Conclusions

Female gender was an independent predictor for 30 day mortality but not for late mortality after surgical treatment of IE. Striking differences regarding sex-specific risk factors indicate that analyses of a mixed male and female cohort may overlook important risk factors. The higher risk for early postoperative mortality in women should not per se lead to avoidance of surgical therapy in women, as surgical treatment was shown to be a protective factor against early mortality in IE in previous studies. Consideration of sex-specific risk factors in prevention, interdisciplinary diagnosis, therapy, and an early postoperative course, such as preoperative elevated CRP level and interval from diagnosis to surgery in female patients, as well as the potential risk of culture-negative IE in male patients, may improve outcomes in women and men.

## Figures and Tables

**Table 1:** Baseline patient characteristics stratified by gender.

Variable	Overall (*n* = 413)	Men (*n* = 308, 74.6%)	Women (*n* = 105, 25.4%)	*p*-Value
Age, years	64 (52;73)	64 (50;73)	65 (57;75)	0.082
Body mass index (kg/m^2^)	25.9 (23.0;29.4)	25.7 (23.0;29.0)	26.1 (23.0;30.8)	0.223
Body mass index > 30 (kg/m^2^)	92 (22.4%)	63 (20.6%)	29 (27.6%)	0.136
Logistic EuroSCORE	27.2 (12.5;49.1)	24.8 (11.7;45.6)	35.7 (14.6;53.6)	0.054
EuroSCORE II	12.1 (5.2;27.3)	11.6 (5.0;25.2)	16.3 (6.1;30.8)	0.127
Co-morbidity				
COPD	50 (12.1%)	37 (12.0%)	13 (12.4%)	0.920
Arterial hypertension	240 (58.1%)	177 (57.5%)	63 (60.0%)	0.650
Pulmonary hypertension (>25 mmHg)	86 (20.9%)	60 (19.5%)	26 (25.0%)	0.231
Atrial fibrillation	81 (19.6%)	61 (19.8%)	20 (19.0%)	0.866
Peripheral vascular disease	36 (8.7%)	28 (9.1%)	8 (7.6%)	0.644
Type 1 Diabetes mellitus	3 (0.7%)	2 (0.6%)	1 (1.0%)	1.000
Type 2 Diabetes mellitus	83 (20.1%)	58 (18.8%)	25 (23.8%)	0.272
IDDM	45 (10.9%)	30 (9.7%)	15 (14.3%)	0.197
Hyperlipoproteinemia	116 (28.1%)	82 (26.6%)	34 (32.4%)	0.257
Dialysis (acute and chronic)	45 (10.9%)	32 (10.4%)	13 (12.4%)	0.572
Acute renal insufficiency	53 (12.8%)	42 (13.6%)	11 (10.5%)	0.403
Chronic dialysis preoperative	18 (4.4%)	11 (3.6%)	7 (6.7%)	0.177
Chronic renal insufficiency	116 (28.1%)	92 (29.9%)	24 (22.9%)	0.167
NYHA IV	83 (20.2%)	65 (21.2%)	18 (17.3%)	0.388
Tumor	55 (13.3%)	40 (13.0%)	15 (14.3%)	0.735
Rheumatic disease	23 (5.6%)	16 (5.2%)	7 (6.7%)	0.570
History of liver disease	55 (13.3%)	42 (13.7%)	13 (12.4%)	0.735
Drug abuse	23 (5.6%)	16 (5.2%)	7 (6.7%)	0.570
Smoking ^1^	103 (27.8%)	78 (28.4%)	25 (26.0%)	0.662
Immunosuppressive therapy	11 (2.7%)	6 (1.9%)	5 (4.8%)	0.156
Previous endocarditis	60 (14.5%)	51 (16.6%)	9 (8.6%)	**0.045**
LVEF poor (<30)	41 (10.5%)	35 (12.0%)	6 (6.1%)	0.096
Coronary heart disease	178 (43.2%)	142 (46.3%)	36 (34.3%)	**0.033**
Single-vessel disease	76 (18.4%)	56 (18.2%)	20 (19.0%)	**0.854**
Two-vessel-disease	36 (8.7%)	29 (9.4%)	7 (6.7%)	**0.384**
Three-vessel disease	66 (16.0%)	57 (18.6%)	9 (8.6%)	**0.016**
Previous cardiac surgery	171 (41.4%)	125 (40.6%)	46 (43.8%)	0.562
Previous CABG	9 (2.2%)	7 (2.3%)	2 (1.9%)	1.000
Aortic valve replacement	69 (16.7%)	45 (14.6%)	24 (22.9%)	**0.050**
Mitral valve replacement/ resection	6 (1.5%)	4 (1.3%)	2 (1.9%)	0.067
Combined valve surgery	79 (19.1%)	63 (20.5%)	16 (15.2%)	0.241
TAVI	2 (0.5%)	2 (0.6%)	0 (0%)	1.000
Clinical presentation				
Acute myocardial infarction (≤48 h)	14 (3.4%)	11 (3.6%)	3 (2.9%)	1.000
Cardiogenic shock	21 (5.1%)	18 (5.8%)	3 (2.9%)	0.229
CPR (≤48 h)	9 (2.2%)	8 (2.6%)	1 (1.0%)	0.449
Emergency	90 (21.8%)	72 (23.4%)	18 (17.1%)	0.181
Transfer from intensive care unit	109 (26.5%)	80 (26.1%)	29 (27.6%)	0.754
Intubated at admission	38 (9.2%)	29 (9.4%)	9 (8.6%)	0.796
Neurological deficits (TIA or stroke)	81 (19.6%)	55 (17.9%)	26 (24.8%)	0.124
Stroke	76 (18.4%)	53 (17.2%)	23 (21.9%)	0.283
Preoperative embolization	114 (27.6%)	81 (26.3%)	33 (31.4%)	0.310
Embolization of several organs	28 (6.8%)	17 (5.5%)	11 (10.5%)	0.081
Fever (≥38 °C)	270 (66.5%)	206 (68.0%)	64 (62.1%)	0.277
Fever until surgery	63 (15.5%)	48 (15.8%)	15 (14.6%)	0.757
Time from diagnosis to surgery >7 days	243 (59.3%)	180 (58.8%)	63 (60.6%)	0.753
Time from antibiotic start to surgery				
≤1 day	59 (14.5%)	49 (16.1%)	10 (9.6%)	0.104
2–3 days	38 (9.3%)	23 (7.6%)	15 (14.4%)	**0.038**
4–7 days	47 (11.5%)	39 (12.8%)	8 (7.7%)	0.157
>7 days	264 (64.7%)	193 (63.5%)	71 (68.3%)	0.378
Pathogens				
*Staphylococcus aureus*	82 (20.0%)	59 (19.3%)	23 (21.9%)	0.562
*Enterococcus*	61 (14.8%)	48 (15.7%)	13 (12.4%)	0.411
*Streptococcus viridans*	43 (10.5%)	35 (11.4%)	8 (7.6%)	0.270
*Gram-positive streptococcus*	37 (9.0%)	26 (8.5%)	11 (10.5%)	0.541
HACEK group	1 (0.2%)	1 (0.3%)	0 (0%)	1.000
Mycosis	6 (1.5%)	3 (1.0%)	3 (2.9%)	0.177
Culture negative IE	113 (27.5%)	85 (27.8%)	28 (26.7%)	0.826
*Staphylococcus epidermidis*	28 (6.8%)	18 (5.9%)	10 (9.5%)	0.201
MRSA	14 (3.4%)	10 (3.3%)	4 (3.8%)	0.760
Affected valves				
Aortic valve endocarditis	168 (40.7%)	137 (44.5%)	31 (29.5%)	**0.007**
Isolated Aortic valve endocarditis	128 (31.0%)	107 (34.7%)	21 (20.0%)	**0.005**
Mitral valve endocarditis	129 (31.2%)	90 (29.2%)	39 (37.1%)	0.130
Isolated Mitral valve endocarditis	92 (22.3%)	61 (19.8%)	31 (29.5%)	**0.039**
Tricuspid valve endocarditis	16 (3.9%)	8 (2.6%)	8 (7.6%)	**0.036**
Isolated Tricuspid valve endocarditis	7 (1.7%)	4 (1.3%)	3 (2.9%)	0.377
Isolated Prosthetic endocarditis	143 (34.6%)	104 (33.8%)	39 (37.1%)	0.530
Paravalvular leak	17 (4.1%)	13 (4.2%)	4 (3.8%)	1.000
Valve insufficiency (at least grade 2)	359 (87.3%)	267 (87.3%)	92 (87.6%)	0.923
Aortic valve	108 (26.3%)	87 (28.4%)	21 (20.0%)	0.090
Mitral valve	78 (19.0%)	55 (18.0%)	23 (21.9%)	0.375
Tricuspid valve	8 (1.9%)	3 (1.0%)	5 (4.8%)	**0.029**
Peri-annular abscess	113 (27.8%)	81 (26.6%)	32 (31.1%)	0.386
Vegetation	285 (70.4%)	209 (69.2%)	76 (73.8%)	0.309
Preoperative laboratory results				
C-reactive protein (mg/L)	42.7 (16.4;90.5)	43.5 (19.3;91.1)	41.2 (13.6;88.2)	0.565

Significant *p*-values are indicated in bold. Quantitative data are presented as median with 25th and 75th percentiles, while categorical data are presented as number of patients (*n*) with percentage (%). Missing values > 5%: ^1^ 10.2% missing. European System for Cardiac Operative Risk Evaluation is abbreviated to EuroSCORE, chronic obstructive pulmonary disease to COPD, insulin dependent diabetes mellitus to IDDM, New York Heart Association heart failure stage to NYHA, left ventricular ejection fraction to LVEF, Coronary artery bypass grafting to CABG, transcatheter aortic valve implantation to TAVI, cardiopulmonary resuscitation to CPR, transient ischemic attack to TIA, *Haemophilus*, *Aggregatibacter*, *Cardiobacterium*, *Eikenella*, *Kingella* to HACEK, methicillin-resistant *Staphylococcus aureus* to MRSA.

**Table 2:** Operative data stratified by gender.

Variable	Overall (*n* = 413)	Men (*n* = 308, 74.6%)	Women (*n* = 105, 25.4%)	*p*-Value
Length of surgery (min)	273 (220;355)	274 (224;357)	271 (216;337)	0.411
Cardiopulmonary bypass time (min)	166 (125;215)	166 (126;214)	166 (121;219)	0.879
Cross-clamp time (min)	116 (86;156)	115 (86;157)	116 (83;144)	0.433
Circulatory arrest (min)	0 (0–36)	0 (0–36)	0 (0–32)	0.520
Number of packed red blood cells (unit)	3 (0–27)	2 (0–27)	4 (0–14)	**<0.001**
Number of fresh frozen plasma (unit)	0 (0–13)	0 (0–13)	0 (0–12)	0.900
Number of platelet concentrate (unit)	1 (0–6)	1 (0–6)	1 (0–4)	0.143
Aortic valve surgery	305 (74.2%)	239 (78.1%)	66 (62.9%)	**0.002**
Mitral valve surgery	155 (37.7%)	110 (35.9%)	45 (42.9%)	0.207
Tricuspid valve surgery	15 (3.6%)	8 (2.6%)	7 (6.7%)	0.070
Thoracic aortic surgery	55 (13.4%)	43 (14.1%)	12 (11.5%)	0.509
CABG	49 (11.9%)	37 (12.1%)	12 (11.4%)	0.856

Significant *p*-values are indicated in bold. Quantitative data are presented as median with 25th and 75th percentiles, while categorical data are presented as number of patients (*n*) with percentage (%).

**Table 3:** Postoperative data and outcomes stratified by gender.

Variable	Overall (*n* = 413)	Men (*n* = 308, 74.6%)	Women (*n* = 105, 25.4%)	*p*-Value
AKI KDIGO stages	115 (29.3%)	85 (29.2%)	30 (29.4%)	0.969
New–onset of hemodialysis	61 (15.6%)	46 (15.8%)	15 (14.9%)	0.819
24 h-drainage loss (mL)	600 (300;1100)	650 (388;1150)	510 (250;1060)	0.123
Rethoracotomy (bleeding/tamponade)	50 (12.4%)	40 (13.3%)	10 (9.7%)	0.341
Number of packed red blood cells (unit) ^1^	2 (0–27)	2 (0–27)	2 (0–17)	**0.015**
Number of fresh frozen plasma, (unit) ^1^	0 (0–35)	0 (0–35)	0 (0–32)	0.269
Number of platelet concentrate, (unit) ^1^	0 (0–9)	0 (0–8)	0 (0–9)	0.357
Ventilation time (h)	16 (9;45)	16 (9;44)	16 (9;55)	0.801
Reintubation	49 (12.3%)	33 (11.1%)	16 (15.7%)	0.220
Tracheotomy	57 (14.5%)	47 (16.2%)	10 (9.9%)	0.125
ICU time (d)	3 (1;7)	3 (1;7)	3 (1;6)	0.245
Postoperative days (d)	10 (7;16)	10 (7;17)	9 (5;15)	**0.046**
Postoperative delirium	64 (16.1%)	54 (18.2%)	10 (10.0%)	0.054
Stroke	18 (4.5%)	11 (3.7%)	7 (6.9%)	0.265
CPR	22 (5.5%)	17 (5.7%)	5 (4.9%)	0.759
Pacemaker patient	47 (11.6%)	37 (12.2%)	10 (9.8%)	0.511
Postoperative myocardial infarction	5 (1.3%)	4 (1.3%)	1 (1.0%)	1.000
Bronchopulmonary infection	45 (11.1%)	36 (11.8%)	9 (8.9%)	0.422
Sepsis	54 (13.3%)	40 (13.1%)	14 (13.9%)	0.839
Sternal wound infection ^2^	9 (2.5%)	8 (2.9%)	1 (1.1%)	0.694
Hospital mortality	68 (16.6%)	45 (14.7%)	23 (22.3%)	0.070
Cardiac death	10 (14.3%)	6 (13.3%)	4 (16.0%)	0.737
Cerebral death	1 (1.4%)	1 (2.2%)	0 (0%)	1.000
Sepsis	9 (12.9%)	5 (11.1%)	4 (16.0%)	0.712
MOF	50 (71.4%)	33 (73.3%)	17 (68.0%)	0.636
30 day mortality	74 (17.9%)	46 (14.9%)	28 (26.7%)	**0.007**
Survival/follow-up time (years)	3.9 (1.2;7.7)	3.7 (1.1;7.8)	4.6 (1.4;7.8)	0.535

Significant *p*-values are indicated in bold. Quantitative data are presented as median with 25th and 75th percentiles, while categorical data are presented as number of patients (*n*) with percentage (%). Missing values > 5%: ^1^ Number of blood products given within 48 hr postoperatively, 5.8% missing, ^2^ 11.1% missing. AKI is abbreviated to acute kidney injury, Kidney Disease: Improving Global Outcomes to KDIGO, intensive care unit to ICU, cardiopulmonary resuscitation to CPR, multiple organ failure to MOF.

**Table 4:** Independent preoperative risk factors for 30 day mortality.

Risk Factors Group	Odds Ratio Overall	95% CI	*p*-Value	Odds Ratio Men	95% CI	*p*-Value	Odds Ratio Women	95% CI	*p*-Value
Age (years)	1.036	1.010–1.063	0.006						
Age ≥ 65 years							4.921	1.048–23.099	0.043
Age ≥ 70 years				2.836	1.265–6.357	0.011			
Female gender	2.090	1.077–4.053	0.029						
Body mass index				1.104	1.026–1.189	0.008			
PH				3.500	1.440–8.508	0.006			
Dialysis				5.943	2.019–17.494	0.001	6.974	1.133–42.922	0.036
NYHA IV	2.719	1.344–5.500	0.005	3.108	1.344–7.189	0.008			
Cardiogenic shock	3.415	1.027–11.350	0.045	9.083	2.418–34.112	0.001			
Stroke	2.664	1.281–5.543	0.009						
Transfer from ICU							10.086	1.791–56.806	0.009
AV insufficiency	0.341	0.133–0.879	0.026						
Fever until surgery				2.828	1.030–7.768	0.044			
Culture negative				2.661	1.161–6.100	0.021			
Abscess	2.513	1.332–4.742	0.004	2.570	1.075–6.142	0.034			
CRP (mg/L)	1.008	1.003–1.012	0.001				1.012	1.002–1.022	0.021
AV endocarditis							0.041	0.004–0.432	0.007
Embolization				4.678	1.032–21.194	0.045			

Pulmonary hypertension > 25 mm Hg is abbreviated to PH, New York heart association heart failure stage to NYHA, intensive care unit to ICU, Aortic valve to AV, C-reactive protein to CRP. Dialysis = Acute and chronic dialysis, Embolization = Embolization of several organs.

**Table 5:** Independent preoperative risk factors for mortality during follow-up.

Risk Factors Group	Hazard Ratio Overall	95% CI	*p*-Value	Hazard Ratio Men	95% CI	*p*-Value	Hazard Ratio Women	95% CI	*p*-Value
Age ≥ 65 years	2.198	1.385–3.488	<0.001	1.921 *	1.287–2.868	0.001	2.066	1.056–4.040	0.034
Female gender	1.097	0.662–1.820	0.718						
AHT				1.685	1.076–2.637	0.022			
NYHA IV							4.192	1.954–8.993	<0.001
Poor LVEF (<30%)	1.945	1.000–3.783	0.050	2.166	1.342–3.497	0.002			
PAD	2.515	1.291–4.900	0.007	1.795	1.023–3.152	0.041			
CAD stage 3							4.040	1.633–9.994	0.003
Dialysis	2.186	1.102–4.338	0.025	1.926	1.145–3.241	0.014	3.383	1.317–8.688	0.011
Combined surgery	2.223	1.236–3.998	0.008	1.709	1.054–2.770	0.030			
Cardiogenic shock				3.601	1.803–7.189	<0.001			
Emergency							5.850	2.439–14.032	<0.001
Diagnosis > 7 days							2.902	1.341–6.282	0.007
Stroke				2.165	1.334–3.516	0.002			
Tumor				1.687	1.014–2.808	0.044			
Liver disease	1.912	1.090–3.355	0.024	2.114	1.310–3.413	0.002			
*S. viridans*				0.336	0.123–0.924	0.035			
*S. epidermidis*							4.878	1.680–14.160	0.004
AV endocarditis							0.300	0.123–0.734	0.008
Abscess				1.561	1.038–2.348	0.032			
CRP (mg/L)							1.006	1.001–1.010	0.018

Peripheral arterial disease is abbreviated to PAD, Arterial hypertension to AHT, New York heart association heart failure stage to NYHA, Coronary heart disease to CAD, *Streptococcus viridans* to *S. viridans*, *Staphylococcus epidermidis* to *S. epidermidis*, Aortic valve to AV, C-reactive protein to CRP, Dialysis = Acute and chronic dialysis, Combined surgery = Combined valve surgery, Diagnosis > 7 days = Diagnosis until surgery > 7 days. * Age in the male group was categorized to ≥ 70 years.

## Data Availability

The data presented in this study are available on request from the corresponding author.

## Supplementary Materials

The following supporting information can be downloaded at: https://www.mdpi.com/article/10.3390/jcm11071875/s1, Table S1: Pre- and intraoperative characteristics associated with 30-day mortality (*p* ≤ 0.1), Figure S1: Flow chart of case numbers over the course of the study.
