# Supplementary material for: Sex-Specific Risk Factors for Short- and Long-Term Outcomes after Surgery in Patients with Infective Endocarditis
Source: J Clin Med. 2022 Mar 28;11(7):1875. doi: 10.3390/jcm11071875 (PMC8999412; doi:10.3390/jcm11071875)
Supplement: Supplementary file 1 [file jcm-11-01875-s001.zip › Table S1.pdf]

Table S1: Pre- and intraoperative characteristics associated with 30-day mortality ($p \leq 0.1$)

	Overall			Men (n=308)			Women (n=105)			Sex-specific interaction
	30-day survivors (n=339, 82%)	30-day non-survivors (n=74, 18%)	*p*-Value	30-day survivors (n=262, 85.1%)	30-day non-survivors (n=46,14,9%)	*p*-Value	30-day survivors (n=77, 73.3%)	30-day non-survivors (n=28, 26.7%)	*p*-Value	*p*-Value
Female gender	77 (2.7%)	28 (37.8%)	**0.007**							
Age, years	63 (50;72)	69 (58;77)	**0.001**	63 (50;72)	69 (51;76)	**0.032**	64 (52;73)	69 (61;77)	**0.025**	
Age ≥ 65 years	157 (46.3%)	47 (63.5%)	**0.007**	121 (46.2%)	27 (58.7%)	0.117	36 (46.8%)	20 (71.4%)	**0.025**	0.347
Age ≥ 70 years	101 (29.8%)	36 (48.6%)	**0.002**	75 (28.6%)	22 (47.8%)	**0.010**	26 (33.8%)	14 (50.0%)	0.130	0.783
Body mass index [kg/m²]	25.7 (22.7;29.4)	27.5 (23.5;29.4)	0.077	25.5 (22.6;28.6)	27.5 (24.0;29.4)	0.064	26.1 (22.7;31.2)	27.0 (23.3;30.2)	0.800	BMI > 30: 0.679
Logistic EuroSCORE I	23.3 (11.1;43.1)	51.3 (27.2;71.9)	**<0.001**	21.3 (11.1;41.6)	48.4 (27.2;76.8)	**<0.001**	26.9 (12.8;46.1)	52.2 (25.7;68.4)	**0.002**	
EuroSCORE II	10.0 (4.2;22.1)	26.8 (14.4;49.8)	**<0.001**	9.7 (4.2;21.5)	23.9 (15.4;53.9)	**<0.001**	12.2 (4.3;25.6)	30.0 (10.2;45.6)	**0.002**	
Pulmonary hypertension[1]	65 (19.2%)	21 (28.8%)	0.067	46 (17.6%)	14 (30.4%)	**0.042**	19 (24.7%)	7 (25.9%)	0.897	0.296
Peripheral arterial disease	26 (7.7%)	10 (13.5%)	0.106	24 (9,2%)	4 (8.7%)	1.000	2 (2.6%)	6 (21.4%)	**0.004**	**0.020**
Type 1 Diabetes mellitus	1 (0.3%)	2 (2.7%)	0.084	0 (0%)	2 (4.3%)	**0.022**	1 (1.3%)	0 (0%)	1.000	
Type 2 Diabetes mellitus	63 (18.6%)	20 (27.0%)	0.101	44 (16.8%)	14 (30.4%)	**0.029**	19 (24.7%)	6 (21.4%)	0.730	0.136
IDDM	32 (9.4%)	13 (17.6%)	**0.042**	22 (8.4%)	8 (17.4%)	0.100	10 (13.0%)	5 (17.9%)	0.538	0.543
Dialysis (acute and chronic)	28 (8.3%)	17 (23.0%)	**<0.001**	21 (8.0%)	11 (23.9%)	**0.003**	7 (9.1%)	6 (21.4%)	0.103	0.704
Acute renal insufficiency	37 (10.9%)	16 (21.6%)	**0.013**	29 (11.1%)	13 (28.3%)	**0.002**	8 (10.4%)	3 (10.7%)	1.000	0.168
Chronic renal insufficiency	91 (26.8%)	25 (33.8%)	0.229	73 (27.9%)	19 (41.3%)	0.066	18 (23.4%)	6 (21.4%)	0.833	0.256
Chronic dialysis preoperative	9 (2.7%)	9 (12.2%)	**0.001**	6 (2.3%)	5 (10.9%)	**0.014**	3 (3.9%)	4 (14.3%)	0.080	0.817
NYHA IV	55 (16.4%)	28 (37.8%)	**<0.001**	46 (17.7%)	19 (41.3%)	**<0.001**	9 (11.8%)	9 (32.1%)	**0.021**	0.907
Liver cirrhosis	5 (1.5%)	5 (6.8%)	**0.020**	5 (1.9%)	4 (8.7%)	**0.032**	0 (0%)	1 (3.6%)	0.267	1.000
LVEF (%), 5.3% missings[2]	55 (50;55)	55 (40;55)	**0.006**	55 (50;55)	55 (27;55)	**0.017**	55 (55;56)	55 (44;55)	0.083	
LVEF poor (<30)	24 (7.5%)	17 (23.9%)	**<0.001**	23 (9.3%)	12 (26.7%)	**0.001**	1 (1.4%)	5 (19.2%)	**0.004**	0.186
CHD: Three-vessel disease	51 (15.1%)	15 (20.3%)	0.271	47 (18.0%)	10 (21.7%)	0.548	4 (5.2%)	5 (17.9%)	0.055	0.160
Previous cardiac surgery	129 (38.1%)	42 (56.8%)	**0.003**	100 (38.2%)	25 (54.3%)	**0.039**	29 (37.7%)	17 (60.7%)	**0.035**	0.611
Combined valve surgery	59 (17.4%)	20 (27.0%)	0.057	50 (19.1%)	13 (28.3%)	0.155	9 (11.7%)	7 (25.0%)	0.124	0.540
Cardiogenic shock	10 (2.9%)	11 (14.9%)	**<0.001**	9 (3.4%)	9 (19.6%)	**<0.001**	1 (1.3%)	2 (7.1%)	0.173	0.907
CPR (≤ 48h)	5 (1.5%)	4 (5.4%)	0.059	5 (1.9%)	3 (6.5%)	0.101	0 (0%)	1 (3.6%)	0.267	1.000
Emergency	67 (19.8%)	23 (31.1%)	**0.033**	57 (21.8%)	15 (32.6%)	0.109	10 (13.0%)	8 (28.6%)	0.080	0.501
Transfer from ICU	78 (23.0%)	31 (42.5%)	**0.001**	63 (24.0%)	17 (37.8%)	0.053	15 (19.5%)	14 (50.0%)	**0.002**	0.189
Intubated at admission	28 (8.3%)	10 (13.5%)	0.157	24 (9.2%)	5 (10.9%)	0.783	4 (5.2%)	5 (17.9%)	0.055	0.178
Stroke	56 (16.5%)	20 (27.0%)	**0.035**	41 (15.6%)	12 (26.1%)	0.084	15 (19.5%)	8 (28.6%)	0.319	0.824
Embolization of several organs	20 (5.9%)	8 (10.8%)	0.128	11 (4.2%)	6 (13.0%)	**0.027**	9 (11.7%)	2 (7.1%)	0.723	0.069
Fever until surgery	48 (14.5%)	15 (20.3%)	0.211	37 (14.4%)	11 (23.9%)	0.104	11 (14.7%)	4 (14.3%)	1.000	0.376
Time from diagnosis to surgery > 7 days	203 (60.4%)	40 (54.1%)	0.313	158 (60.8%)	22 (47.8%)	0.100	45 (59.2%)	18 (64.3%)	0.639	0.186

Time from antibiotic start to surgery < 7 days	114 (34.1%)	30 (40.5%)	0.297	88 (34.1%)	23 (50.0%)	**0.039**	26 (34.2%)	7 (25.0%)	0.371	0.063
**Pathogens**										
Staphylococcus aureus	60 (17.8%)	22 (29.7%)	**0.020**	46 (17.7%)	13 (28.3%)	0.094	14 (18.2%)	9 (32.1%)	0.126	0.808
Streptoccus viridans	42 (12.5%)	1 (1.4%)	**0.005**	35 (13.5%)	0 (0%)	**0.008**	7 (9.1%)	1 (3.6%)	0.679	0.998
Grampos. Streptococcus	36 (10.7%)	1 (1.4%)	**0.011**	25 (9.6%)	1 (2.2%)	0.147	11 (14.3%)	0 (0%)	**0.034**	0.999
Culture negativeIE	87 (25.8%)	26 (35.1%)	0.104	66 (25.4%)	19 (41.3%)	**0.026**	21 (27.3%)	7 (25.0%)	0.816	0.163
Isolated aortic valve IE	115 (33.9%)	13 (17.6%)	**0.006**	95 (36.3%)	12 (26.1%)	0.181	20 (26.0%)	1 (3.6%)	**0.011**	0.111
Aortic valve IE	148 (43.7%)	20 (27.0%)	**0.008**	120 (45.8%)	17 (37.0%)	0.266	28 (36.4%)	3 (10.7%)	**0.011**	0.103
Isolated prosthetic endocarditis	111 (32.7%)	32 (43.2%)	0.085	86 (32.8%)	18 (39.1%)	0.404	25 (32.5%)	14 (50.0%)	0.100	0.411
Prosthetic endocarditis	121 (35.7%)	38 (51.4%)	**0.012**	92 (35.1%)	23 (50.0%)	0.054	29 (37.7%)	15 (53.6%)	0.144	0.952
Aortic valve insufficiency [3]	99 (29.3%)	9 (12.3%)	**0.003**	79 (30.3%)	8 (17.8%)	0.086	20 (26.0%)	1 (3.6%)	**0.011**	0.169
Peri-annular bscess	77 (23.1%)	36 (48.6%)	**<0.001**	59 (22.9%)	22 (47.8%)	**<0.001**	18 (24.0%)	14 (50.0%)	**0.011**	0.967
Combined surgery [4]	153 (45.1%)	40 (55.6%)	0.108	118 (45.0%)	28 (63.6%)	**0.022**	35 (45.5%)	12 (42.9%)	0.813	0.122
**Preoperative laboratory**										
CRP (mg/L)	37.0 (14.6;76.1)	76.6 (44.9;141.6)	**<0.001**	37.7 (16.0;76.0)	84.0 (46.4;138.3)	**<0.001**	26.8 (11.9;77.7)	66.4 (43.0;162.0)	**0.001**	CRP > 40: 0.739

Significant *p*-values are indicated in bold. Quantitative data are presented as median with 25th and 75th percentiles, while categorical data are presented as number of patients (n) with percentage (%). [1] Pulmonary hypertension: sPAP >25 mmHg, [2] Missing values > 5%, [3] at least grade 2, medium, [4] with CABG, ventricular septal defect/atrial septal defect-closure or other. **EuroSCORE**: European System for Cardiac Operative Risk Evaluation; **IDDM**, insulin dependent diabetes mellitus; **NYHA**, New York Heart Association heart failure stage; **LVEF**, left ventricular ejection fraction; **CHD**, coronary heart disease; **CPR**, cardiopulmonary resuscitation; **ICU**, intensive care unit; **CRP**, C-reactive protein
