# Supplementary figures and images for: Sex-Specific Risk Factors for Short- and Long-Term Outcomes after Surgery in Patients with Infective Endocarditis
Source: J Clin Med. 2022 Mar 28;11(7):1875. doi: 10.3390/jcm11071875 (PMC8999412; doi:10.3390/jcm11071875)

Figure S1: Flow chart of case numbers over the course of the study.

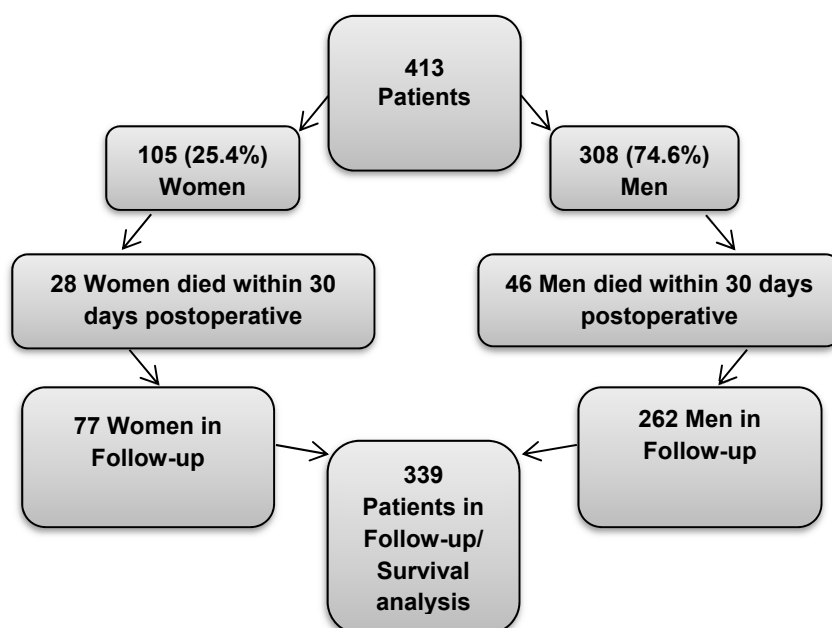

Supplement: Supplementary file 1 [file jcm-11-01875-s001.zip › Figure S1.pdf]
